# Acute Induction of Translocon-Mediated Ca^2+^ Leak Protects Cardiomyocytes Against Ischemia/Reperfusion Injury

**DOI:** 10.3390/cells9051319

**Published:** 2020-05-25

**Authors:** Ribal Al-Mawla, Mallory Ducrozet, Nolwenn Tessier, Lucille Païta, Bruno Pillot, Yves Gouriou, Camille Villedieu, Zeina Harhous, Alexandre Paccalet, Claire Crola Da Silva, Michel Ovize, Gabriel Bidaux, Sylvie Ducreux, Fabien Van Coppenolle

**Affiliations:** 1 Univ Lyon, CarMeN Laboratory, INSERM, INRA, INSA Lyon, Université Claude Bernard Lyon 1, 69500 Bron, France; ribal.al-mawla@hotmail.com (R.A.-M.); mallory.ducrozet@gmail.com (M.D.); nolwenn.tessier@icm-mhi.org (N.T.); lucille.paita@gmail.com (L.P.); bruno.pillot@univ-lyon1.fr (B.P.); yves.gouriou@univ-lyon1.fr (Y.G.); camille.villedieu@gmail.com (C.V.); zeina.harhous@gmail.com (Z.H.); alexandre.paccalet@univ-lyon1.fr (A.P.); claire.crola-da-silva@univ-lyon1.fr (C.C.D.S.); Michel.Ovize@chu-lyon.fr (M.O.); gabriel.bidaux@univ-lyon1.fr (G.B.); fabien.van-coppenolle@univ-lyon1.fr (F.V.C.); 2 Hospices Civils de Lyon, Groupement Hospitalier EST, Département de Cardiologie, IHU-OPERA Bâtiment B13, 69500 Bron, France; 3 Cardiovascular functional explorations, Louis Pradel hospital, Hospices Civils de Lyon, 69677 Lyon, France

**Keywords:** Ca^2+^ leak channel, translocon, reticulum, cardioprotection, ischemia-reperfusion

## Abstract

During myocardial infarction, dysregulation of Ca^2+^ homeostasis between the reticulum, mitochondria, and cytosol occurs in cardiomyocytes and leads to cell death. Ca^2+^ leak channels are thought to be key regulators of the reticular Ca^2+^ homeostasis and cell survival. The present study aimed to determine whether a particular reticular Ca^2+^ leak channel, the translocon, also known as translocation channel, could be a relevant target against ischemia/reperfusion-mediated heart injury. To achieve this objective, we first used an intramyocardial adenoviral strategy to express biosensors in order to assess Ca^2+^ variations in freshly isolated adult mouse cardiomyocytes to show that translocon is a functional reticular Ca^2+^ leak channel. Interestingly, translocon activation by puromycin mobilized a ryanodine receptor (RyR)-independent reticular Ca^2+^ pool and did not affect the excitation–concentration coupling. Second, puromycin pretreatment decreased mitochondrial Ca^2+^ content and slowed down the mitochondrial permeability transition pore (mPTP) opening and the rate of cytosolic Ca^2+^ increase during hypoxia. Finally, this translocon pre-activation also protected cardiomyocytes after in vitro hypoxia reoxygenation and reduced infarct size in mice submitted to in vivo ischemia-reperfusion. Altogether, our report emphasizes the role of translocon in cardioprotection and highlights a new paradigm in cardioprotection by functionally uncoupling the RyR-dependent Ca^2+^ stores and translocon-dependent Ca^2+^ stores.

## 1. Introduction

During cardiac infarction, ischemia triggers a molecular disaster comprising drop in ATP concentration [1], acidosis [2], reactive oxygen species (ROS) accumulation [3], and Ca^2+^ homeostasis perturbations [4,5,6]. This leads to irreversible cellular damage [7] that can ultimately end by cardiomyocytes (CM) death [8]. In this molecular storm, Ca^2+^ ion, a preponderant second messenger, behaves as key inducer of ischemia/reperfusion (I/R)-mediated cell death. Indeed, the concomitant massive Ca^2+^ leak from internal stores overloads mitochondrial matrix and triggers the mitochondrial permeability transition pore (mPTP) opening [9,10], driving mitochondria to its terminal fate. In resting cardiac muscle, reticular Ca^2+^ concentration is finely tuned by calcium-binding proteins [11] and by the dynamic balance between Ca^2+^ uptake and Ca^2+^ release. Sarco-endoplasmic reticulum Ca^2+^ ATPase (SERCA) is the only known Ca^2+^ uptake pump, whereas Ca^2+^ release channels are divided into two categories: (1) channels activated by signalization pathways such as inositol-tri-phosphate receptors (IP_3_R) and ryanodine receptors (RyR), activated by IP_3_ and the Ca^2+^-induced Ca^2+^-release (CICR) mechanism, respectively, and (2) the Ca^2+^ leak channels, which generate reticular Ca^2+^ leak as a side effect of their own activity [12,13]. The physiological role of reticular Ca^2+^ channels from the first category is already well established, whereas the functionality of the leak channels in cardiomyocytes and their involvement in I/R-mediated mechanisms remain poorly understood. Over the last decade [14], most of the cardioprotective strategies have targeted calcium-mediated cyclophilin D activation in mitochondria [9,15] or the Ca^2+^ transfer from the sarco-endoplasmic reticulum (SR/ER) via IP_3_R_2_ [9,16] or RyR_2_ [4] to the mitochondria. Related preclinical in vitro studies were promising. Unfortunately, they have led to disappointing results in clinical trials [17]. There is thus an urgent need to find out new targets to prevent CM cell death and deleterious consequences of myocardial infarction. 

Remarkably, the translocon (TLC), a component of the translation machinery, contributes to the SR/ER Ca^2+^ leak; meanwhile, it serves as the major entry site of newly synthesized polypeptides in the reticular membrane [18]. Reticular transmembrane proteins Sec61, Sec62, and Sec63 comprise the key component of the protein translocation machinery [19], and trimers of Sec61 α, β, and γ subunits form an aqueous central pore ranging from 2.6 nm to 6 nm [20]. As it represents one of the largest pores in the reticular membrane, its aperture should be tiny controlled to maintain reticular Ca^2+^ homeostasis. To compare, RYR channel shows a narrower diameter, from 0.7 nm to 1 nm [21,22,23]. In absence of translation, GRP78/BiP, a luminal ATP-binding partner of the heat shock protein 70 (HSP70) family of chaperones, binds to the channel to fold neosynthetized proteins and seals the pore [24] to avoid Ca^2+^ leakage [25]. During translation, the nascent polypeptidic chain may trigger a Ca^2+^ leak. In fact, a gap between Sec61 and the ribosomes has been suggested [26,27], allowing Ca^2+^ to cross the channel. In addition, at the end of translation, the polypeptidic chain is no longer in the pore. At that time, ribosomes are still bound and a physiological Ca^2+^ leak occurs [28]. 

Research on the TLC complex is made difficult by its imperative requirement for life, explaining why not so long ago most of the studies to investigate the role of TLC in physiopathology have relied on a tightly adjusted pharmacological modulation [29]. Several works, including ours, have thus demonstrated that TLC is a crucial passive Ca^2+^ leak channel [25,30,31,32,33,34,35,36,37]. More recently, gene silencing experiments have clarified the relative roles of the different Sec proteins, which are described in detail elsewhere [38]. In particular, Linxweiler et al. showed that SEC62 silencing intensifies the reticular Ca^2+^ efflux [39]. Moreover, Lu et al. have observed a diminished cell viability and an extended rate of apoptosis in the human glioblastoma cells when silencing the SEC61G gene (coding for Sec61γ) [40]. Not surprisingly, the term “Sec61 channelopathies” has newly emerged to design diseases directly affecting Sec61 subunits or components implicated in the pore gating (for a review, see [41]).

Given that TLC is ubiquitously expressed [18] and can contribute to cellular fate, we wondered whether this channel could be an effective target to minimize CM cell death during myocardial infarction. Our hypothesis was that acute pharmaceutical activation of TLC could pre-drain reticular Ca^2+^ stores before ischemia. This could prevent the massive cytoplasmic Ca^2+^ overload at reperfusion, as well as the subsequent mitochondrial Ca^2+^ overload, and consequently curtail I/R-mediated cell death.

## 2. Materials and Methods

All chemicals and fluorescent probes were purchased from Sigma-Aldrich and Life Technologies unless otherwise specified.

### 2.1. Animals 

Experiments were carried out on 118 male C57BL/6J mice, aged between 8 to 12 weeks, which were obtained from Charles River laboratories. They received human care conformed to the Guide for the Care and Use of Laboratory Animals in our platform (agreement number C-693880502). 

All our procedures were approved by the local institutional animal research committee (N°BH2012-65 for the chirurgical procedure and N°BH2012-64 for heart collection; date of approval: 3 December 2012). Animals were randomly distributed towards different experiments and surgical procedures (Ca^2+^ measurements: 45; H/R: 4; flow cytometry: 15; I/R: 38; heart rate and blood pressure: 12; protein expression: 4).

### 2.2. CM Isolation

As described previously [9], mice were first injected intraperitoneally by 100 μL of 50 UI/kg heparin sodium and then anesthetized with 70 mg/kg pentobarbital sodium. Once pedal pinch reflexes were completely inhibited, a thoracotomy was performed, and the heart was collected and cannulated by the aorta in a Langendorff system. Blood was washed out with perfusion buffer (in mM: 113 NaCl, 4.7 KCl, 0.6 KH_2_PO_4_, 0.6 Na_2_HPO_4_, 1.2 MgSO_4_-7H_2_O, 0.032 phenol red, 12 NaHCO_3_, 10 KHCO_3_, 10 HEPES (4-(2-hydroxyethyl)-1-piperazineethanesulfonic acid), 30 taurine, 10 mM 2.3-butanedione monoxime, 5.5 mM glucose) and pH was adjusted to 7.4. The heart was washed out with this buffer for 5 min at 37 °C. 

Then, the heart was perfused at 37 °C with a digestion buffer (perfusion buffer 1×, 0.167 mg/mL Liberase Research Grade (Roche), 0.14 mg/mL trypsine 2.5% 10×, 12.5 μM CaCl_2_) at a constant rate. At the end of digestion, the enzymatic activity was interrupted by a stopping buffer 1 (Perfusion Buffer 1×, 10% bovine calf serum, 12.5 μM CaCl_2_) at 4 °C. The left ventricle was isolated in order to detach CM. The solution was then filtered (SEFAR Nitex 102 cm, Zurich Dutsher) in a 10 mL tube. After CM sedimentation, the medium was carefully replaced and cells were re-suspended in stopping buffer 2 (same composition of stopping buffer 1 but with only 5% bovine calf serum). This step was repeated in stopping buffer 2 containing gradually increased Ca^2+^ concentration (stopping buffer 2 + 0.05, 0.1, 0.5, and 1 mM CaCl_2_ by increasing order). At the end of the final incubation, cells were suspended in a M199 medium (Gibco) supplemented with 100 U/mL penicillin, 100 μg/mL streptomycin, and ITS (insulin 1 μg/mL, transferin 0.55 μg/mL, selenium 0.5 ng/mL).

CM were then seeded on 35 mm Ibidi dishes (Ibidi Biovalley; for Ca^2+^ measurements and H/R experiments) or Lab-tek chamber slides (Merck Millipore; for immunostaining), or else on glass coverslips (for Ca^2+^ transients) precoated with 10 μg/mL laminin (Corning), and then incubated for 2 h at 37 °C. Experiments were realized at the same day of the isolation with a 70–80% living CM estimated by observation.

### 2.3. Adenovirus Injection for Reticular and Mitochondrial Ca^2+^ Measurements

Seven- to nine-week-old C57BL/6J mice were anesthetized with isoflurane 2% and buprenorphine (IP, 0.075 mg/kg). Mice oral intubation was performed using a 22-gauge vinyl catheter and ventilated via a mice ventilator (model 687, Harvard Apparatus) with the following parameters: 0.2 mL tidal volume and 160 breaths/min breathe rate. Monitoring of body temperature was realized thanks to a rectal thermometer and maintained at 37 °C using a heating pad. A left thoracotomy was performed in the fourth left intercostal space. When the pericardium was opened, the heart was exposed to perform 4–5 intramyocardiac injections of 5 × 10^8^ PFU (Plaque Formation Unit) of the adenoviruses D4ER (reticular Ca^2+^ sensor) or 4mtD3CPV (mitochondrial Ca^2+^ sensor) in a total volume of 20 μL. After surgery, animals were allowed to recover from anesthesia, and once spontaneous breathing resumed, we removed the endotracheal tube. Seven days after recovery, mice were premedicated intraperitoneally with heparin (100 USP (United States Pharmacopeia) units per mouse). Anesthesia was induced with sodium pentobarbital (70 mg/kg). A thoracotomy was performed and the heart was collected. Adult ventricular cardiomyocytes were isolated using enzymatic digestion as described above. 

### 2.4. Ca^2+^ Measurements

After the 2 h incubation in Ibidi dishes at 37 °C, the culture medium was replaced by a Ca^2+^-containing buffer (CCB; in mM: 140 NaCl, 5 KCl, 10 HEPES, 1 MgCl_2_, 2 CaCl_2_, 10 glucose; adjusted to pH 7.4). Cardiomyocytes were loaded in CCB containing 5 µM of fura-2-acetoxymethyl ester (fura2-AM) (for cytosolic Ca^2+^ imaging) for 30 min at room temperature.

After the fura2-AM loading, cells were washed twice for 5 min with a Ca^2+^-free buffer (CFB) (same as CCB but without CaCl_2_) containing 0.1 mM EGTA (ethylene glycol tetraacetic acid).

Pretreatment protocol: CM were pretreated with 200 µM puromycin for 30 min while being loaded with the probe, and in the case of double pretreatment with emetine, 20 µM of the latter was added 30 min before CM probe loading. In both cases, once the drug was added, it remained present during the whole experiment. The TLC inhibitor (emetine) was applied 5 minutes before its activator (puromycin) to ensure prior TLC inhibition and to avoid concomitant opposite effects of these compounds on TLC Ca^2+^ leakage [31,32]. After signal stabilization, CM were stimulated by either 5 µM ionomycin, 25 µM FCCP (Carbonyl cyanide 4-(trifluoromethoxy)phenylhydrazone), or 10 mM caffeine.

An ischemia-like hypoxia experiment was performed in a hypoxic chamber (Okolab-Bold line) where temperature and oxygen levels were monitored. Fura2-AM-loaded CM were washed with CCB, then, after fluorescence signal stabilization, we replaced the medium to hypoxic buffer (CCB without glucose) deprived from any supplementation or growth factor necessary for CM survival and normal function, and ventilated it with 99% N_2_ in order to replace all oxygen traces in the medium. Oxygen level was decreased in the chamber to 1%. After 30 min of ischemia-like hypoxia, CM were stimulated by 10 mM caffeine.

Measurements were performed using a wide-field LeicaDMI6000B microscope equipped with an Orca-Flash4.0 digital camera (HAMAMATSU). Using a Lambda DG-4+ filter (Sutter instruments), fura-2 AM was excited at 340 and 380 nm and their respective emitted fluorescence lights were measured at wavelength 510 nm. D4ER and 4mtD3CPV (Förster resonance energy transfer (FRET)-based sensors) were excited at 477 nm (CFP: Cyan Fluorescent Protein) and 514 nm (YFP: Yelllow Fluorescent Protein). Their emitted fluorescent lights were assessed at wavelength 540 nm. Images (2048 × 2048 pixels) were taken at 3 second intervals. Free Ca^2+^ content was estimated by the YFP to CFP fluorescence ratio, which was figured out as described in [42]. 

All experiments were performed at room temperature in a calcium-free buffer to prevent capacitative Ca^2+^ entry that would then add to the emptying of intracellular Ca^2+^ stocks, except for in vitro H/R and paced experiments.

### 2.5. Ca^2+^ Transients

For Ca^2+^ transients experiments, CM were plated on 24 mm glass coverslips. Coverslips were mounted on a Quick Change Chamber (RC-47FSLP Warner Instruments) and stimulation was delivered by a MyoPacer Field Stimulator (IonOptix). CM were loaded at 37 °C with fluo5-AM (5 μM) for 30 min. After loading, cells were washed in CCB, then the medium was replaced by a field stimulation buffer (FSB; in mM: 150 NaCl, 5.4 KCl, 10 HEPES, 2 MgCl_2_, 1 glucose, 2.5 pyruvate, 5 creatine, 5 taurine, 2 CaCl_2_). Cytosolic Ca^2+^ transients were recorded in fluo5-AM-loaded CM, field-stimulated at 1 Hz with a current pulse delivered via 2 platinum electrodes at RT for 0.5 ms and at 40 V amplitude. To measure mitochondrial Ca^2+^ transients, we injected mice with 4mtD3cpv adenovirus, as described previously. Isolated mice CM expressing the probe were field-stimulated for 1 min successively at 0.5, 1, and 2 Hz in FSB. 

Fluo5-AM images were acquired with a Nikon A1Rplus confocal microscope equipped with a ×40 oil-immersion objective (line-scan mode = 256 lines per ms). Scanning was performed along the long axis of the cell. Fluo5-AM was excited at 488 nm by argon-ion laser. The respective emitted fluorescent light was collected at wavelength 525/50 nm using one high sensitive GaAsp detector. Cytoplasmic Ca^2+^ signal analysis was performed using custom-written [43] for the amplitude and the rate of decay, and the rising slope was obtained with an exponential fit (Origin Pro 8 software). 4mtD3cpv fluorescence was acquired as above.

### 2.6. Calcein Cobalt Protocol

Calcein method with cobalt quenching was used in order to evaluate mitochondrial mPTP opening [44,45]. Kinetics of mPTP opening were measured in live CM. Briefly, 1 µM calcein-AM was used for cell loading in CCB for 15 min at room temperature. After two washes, cells were additionally incubated for 30 min with 2 mM cobalt chloride and 200 µM sulfinpyrazon in CCB. Using a Nikon confocal microscope, cells were imaged every 5 seconds under resting conditions for 2 min before application of 2 µM ionomycin, which triggered the mPTP opening. Calcein-AM was excited at 488 nm by argon-ion laser. The respective emitted fluorescent light was collected at wavelength 525/50 nm using one high sensitive high sensitive GaAsp detector. A decrease in mitochondrial calcein-AM fluorescence reflects the opening of mPTP. For data analysis, background was subtracted and curves were normalized with the basal fluorescence.

### 2.7. In Vivo Model of Acute myocardial I/R Injury

As described previously, after anesthesia with 0.075 mg/kg of buprenorphine and 70 mg/kg of pentobarbital, C57BL/6 J (male, 8–10 weeks old) mice were intubated and put under assisted respiration. Heart rate was monitored by an electrocardiogram. A thoracotomy was performed, allowing access to the heart after cutting the fifth rib, as well as upper and lower intercostal muscles. The pericardium was then opened in order to place a knot around the anterior interventricular artery (IVA). Ischemia was affirmed on the electrocardiogram by the shift of the ST-segment (flat isoelectric section on electrocardiography between the end of the S wave and the beginning of the T wave) and lasted 45 min. Preconditioning was performed by IV injection of puromycin in the jugular vein 10 min before ischemia, whereas control mice were injected with the same volume of saline solution at 0.9%. At the end of the ischemia, the knot was loosened to reestablish blood circulation and the wound was stitched. Animal state and recovery were monitored for 24 h.

After 24 h of reperfusion, the animal was again anesthetized following the same protocol and then intubated with the rib cage being re-opened. The knot placed around the anterior inter-ventricular artery was recovered, and was then constricted to occlude the artery again. Evans blue dye was then injected through the vena cava, thus allowing the healthy zone to be colored in blue, leaving the ischemic area in pink, the area-at-risk (AR). The diffusion of Evans blue made it possible to discriminate the healthy zone (non-ischemic) of the AR.

Slices of the left ventricle that were 1mm thick were cut from the apex and delicately covered by a glass plate to be photographed. The slices were then incubated for 15 min in triphenyltetrazolium chloride (TTC) in order to discriminate the necrosis area (AN). Slices were weighed and sizes of different areas were determined by computer in planimetry (SigmaScanPro5). Myocardial infarct size was expressed as percentage of the ratio of the necrosis area (AN) over the area-at-risk (AR). Sham mice that underwent surgery without I/R were used as controls.

### 2.8. CM Mortality

Adult mouse cardiomyocytes (250 µg protein) were kept in suspension according to the two experimental groups: hypoxia-reoxygenation group (HR) and preconditioning by puromycine (PreC Puro, 200 µM, 30 min prior to hypoxia reoxygenation sequence), as previously described [46]. HR cells were incubated with 1.5 mL hypoxia buffer (140 mM NaCl, 5 mM KCl,1 mM MgCl_2_, 10 mM HEPES, 2 mM CaCl_2_). Hypoxia cells were transferred into a hypoxia incubator on the rocking platform at 37 °C with 0.5% O_2_. Cells were submitted to hypoxia for 1 h 30 min. In terms of reoxygenation, at the end of hypoxia, medium was changed for plating medium (MEM (Minimal Essential Medium) supplemented with 10% FBS, 1% penicillin/streptomycin, 10 mM butanedione monoxime, 2 mM glutamine, 200 mM ATP) and replaced into the incubator at 37 °C for 1 h. After the total sequence of hypoxia reoxygenation, a Fortessa X-20 instrument (BD Biosciences) was immediately used for flow cytometry measurements. To evaluate the cell viability, cardiomyocytes were loaded with propidium iodide (1 µg/mL) and analyzed extemporaneously. DIVA Software (BD Biosciences) was employed for data analysis by acquisition of 1000 events, and results were expressed as percentage of propidium iodide-positive cells (dead cells).

### 2.9. Statistical Analysis

Data are presented as medians. For small datasets (n < 10), data are represented as scatter plots that also show median. For larger datasets, data are represented as box and whisker plots where the line in the middle of the box is the median, the whiskers are drawn down to the 10% quartile value and up to the 90% quartile value, and additionally a “+” appears at the mean. For comparison between two groups, the Mann–Whitney non-parametric test was used, whereas the non-parametric Kruskal–Wallis test followed by the Dunn’s multiple comparisons test was used to analyze differences between multiple groups (unless otherwise specified). For flow cytometry analysis, Wilcoxon matched-pairs signed rank tests were performed. In the two-way ANOVA, one factor was the effect of puromycin pretreatment and the other factor was the effect of the frequency stimulation; values were matched and Bonferroni post-hoc test was used. Experiments were performed at least three times or more. Statistical analysis was performed with GraphPad Prism 6 software (La Jolla, CA, USA). *p* < 0.05 was considered significant.

## 3. Results and Discussion

### 3.1. TLC Is a Functional Reticular Ca^2+^ Leak Channel in Isolated Mouse CM: Its Activation Mobilized a RyR-Independent Ca^2+^ Reticular Pool and Did Not Affect Excitation–Contraction (E-C) Coupling

**Figure 1:**
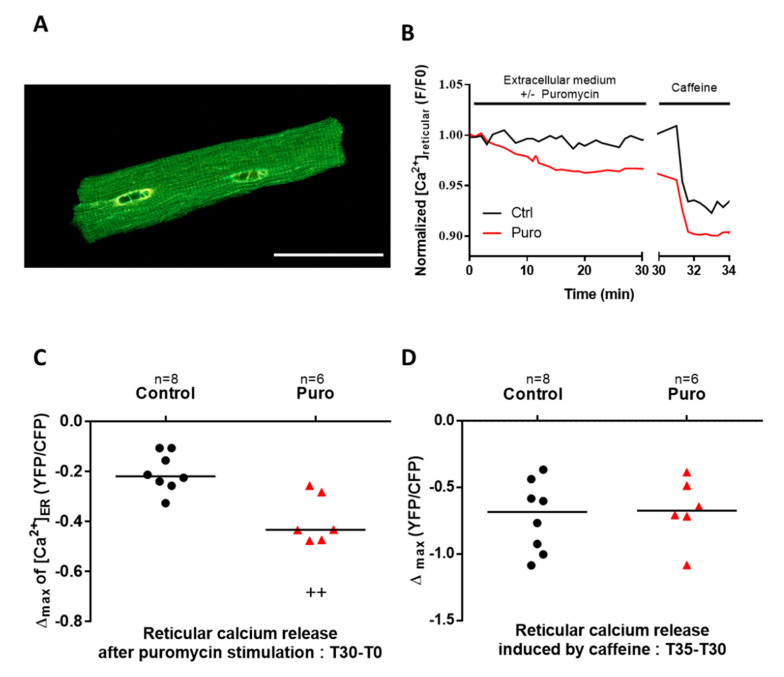
Translocon (TLC) activation by puromycin induces a decrease in reticular Ca^2+^ stock. (**A**) Illustration of the in vivo adenoviral strategy to express a reticular Ca^2+^ sensor, D4ER, in adult mouse cardiomyocytes, presenting a typical reticular pattern of the fluorescent sensor as displayed on the representative confocal image. Scale bar: 50 µm. (**B**) Graphical representation of the D4ER fluorescence ratio evolution with time in control condition (black line) and in response to 30 min of 200 µM puromycin treatment (red line), both stimulated with 5 mM caffeine; representative curve of reticular fluorescence evolution in control (Ctrl) condition and under puromycin treatment. (**C**) Scatter plots of reticular Ca^2+^ decreased at the end of 30 min perfusion with or without puromycin. (**D**) Scatter plots of the ryanodine receptor (RyR)-dependent reticular Ca^2+^ stock after puromycin or Ctrl treatment estimated by caffeine stimulation, calculated as difference between fluorescence level at stimulation time and final fluorescence. *n* = cell count. Statistics: ^++^ *p* < 0.01 vs. Ctrl.

First, TLC expression in mouse CM was confirmed by Western blot (Figure S1). We then investigated the functionality of TLC as a reticular Ca^2+^ leak channel by monitoring the evolution of the reticular Ca^2+^ concentration ([Ca^2+^]_r_). To this end, we used an in vivo adenoviral delivery strategy based on an intramyocardial injection of an adenovirus encoding a FRET-based reticular Ca^2+^ sensor, D4ER (Figure 1A). One week after the injection, adult mouse CM were freshly isolated. These primary cells provided us with a powerful model for heart research at the cellular level. Unfortunately, their lifetime is not compatible with transgenic modulation of TLC component expression, as both require transfection and a significant period of expression. Therefore, our present investigation was based on the use of TLC pharmacological agents. Puromycin mimics the 3’ end of aminoacetylated tRNA (transfer ribonucleic acid), releases the nascent peptidic chain, and allows Ca^2+^ to leak through the pore from the lumen to the cytoplasm [25,31,32]. On the other hand, emetine prevents ribosome binding to TLC and then avoids Ca^2+^ leak [47]. When cardiomyocytes were treated with puromycin (200 µM) for 30 min, we observed a significant and progressive [Ca^2+^]_r_ decrease (Figure 1B,C; fluorescent ratio: −0.4334 vs. −0.2197) showing that TLC is a functional reticular Ca^2+^ leak channel in this model. The reticular RyR-dependent Ca^2+^ pool was then determined by a treatment with caffeine and was similar in control and in puromycin-treated CM (Figure 1D). Interestingly, these findings indicate that the caffeine-sensitive Ca^2+^ stores could be independent of the puromycin-sensitive Ca^2+^ stores, that is, RyR and TLC activation would mobilize two different Ca^2+^ pools. With a comparable approach using an angiotensin II treatment, we found no impact of TLC activation on the IP_3_R-dependant Ca^2+^ reticular stocks (Figure S2). 

**Figure 2:**
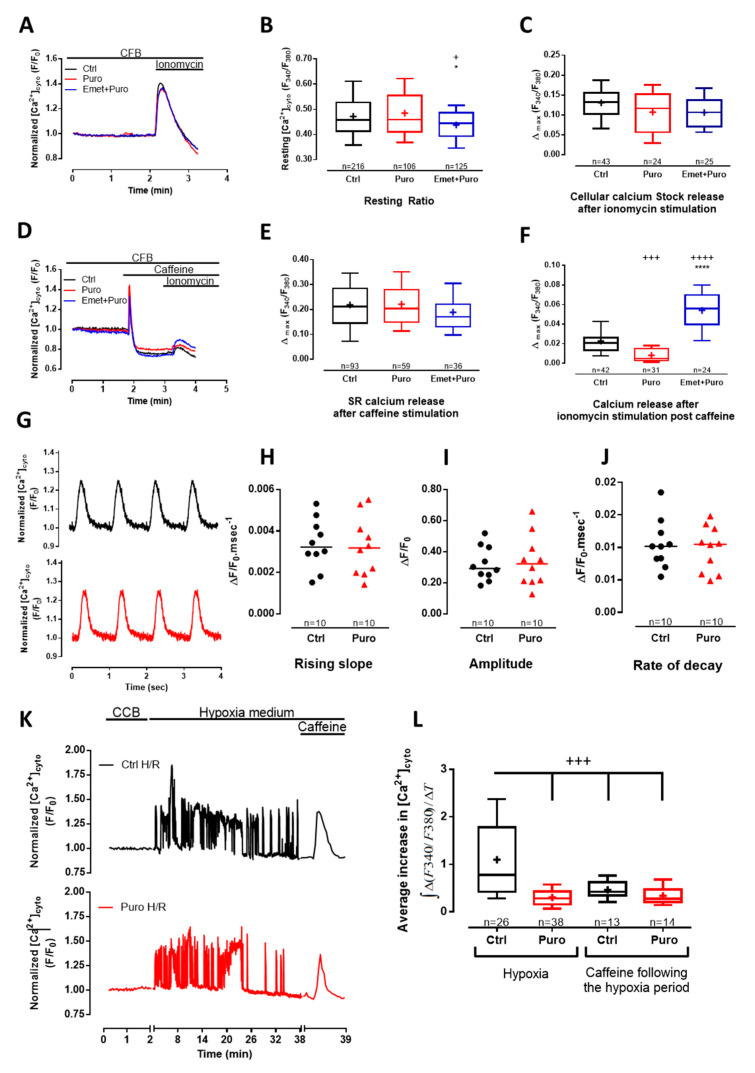
The reticular puromycin-mediated Ca^2+^ stock release was independent of the one stimulated by caffeine and did not affect excitation–contraction (E-C) coupling. (**A**,**D**) Time traces showing cytosolic Ca^2+^ concentration assessed by fura2-AM (acetoxymethyl ester) cytosolic probe, in Ctrl condition and after 30 min of 200 µM puromycin pretreatment or 20 µM emetine +200µM puromycin pretreatment. Effect of puromycin and emetine + puromycin was measured after cardiomyocyte (CM) pretreatment in Ca^2+^-containing buffer (CCB) for 30 min (see the Materials and Methods section). Ca^2+^ content was figured out by the maximum amplitude of fura2-AM fluorescence ratio (∆max Ratio_(340/380)_) after the addition of different stimulations in a Ca^2+^-free buffer (CFB). (**B**) Box blots representing the steady-state cytosolic Ca^2+^ concentration and (**C**) total cell Ca^2+^ content assessed by 5 µM of ionomycin stimulation. (**E**) RyR_2_-dependent Ca^2+^ stores assessed by 10 mM of caffeine stimulation and (**F**) remaining cell Ca^2+^ content after caffeine stimulation assessed by ionomycin stimulation. (**G**–**J**) Cytoplasmic Ca^2+^ transients were recorded using fluo5-AM–loaded intact CM electrically stimulated at 1 Hz. (**G**) Representative cytoplasmic Ca^2+^ transients in the absence or after 30 min of puromycin pretreatment; ΔF/F0 = normalized change fluorescence. (**H**) Scatter blots representing cytoplasmic Ca^2+^ transients amplitude, (**I**) Ca^2+^ transients rising slope (calculated from the relative amplitude and time to peak of the electrical induced Ca^2+^ transient), and (**J**) Ca^2+^ transients rate of decay. (**K**) Time traces displaying cytosolic Ca^2+^ concentration (measured by the fura2-AM fluorescence ratio) in a Ctrl CM and in a 200 µM puromycin preconditioned CM subjected to a 30 min ischemia-like hypoxia. At the end of the 30 min, 10 mM caffeine was added to the medium. CCB means Ca^2+^-containing buffer. (**L**) Average increase in the cytosolic Ca^2+^ concentration [Ca^2+^]_cyto_ was figured out as masses of fura2-AM fluorescence signal over time in CM treated as explained in (**K**). Data are from at least three independent experiments. *n* = cell count. Statistics: + *p* < 0.05, +++ *p* < 0.001, ++++ *p* < 0.0001 vs. Ctrl, * *p* < 0.05, **** *p* < 0.0001 vs. Puro (puromycin).

**Figure 3:**
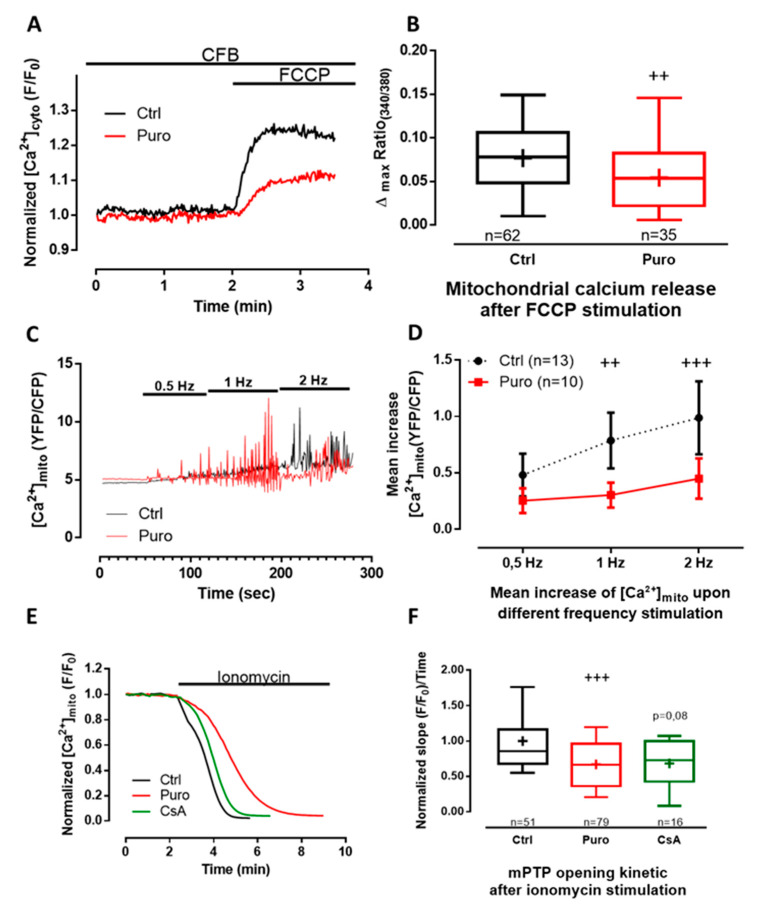
Puromycin pretreatment modified mitochondrial Ca^2+^ content in beating CM and delays mitochondrial permeability transition pore (mPTP) opening. (**A**) Representative time traces of mitochondrial Ca^2+^ concentration (expressed as the 340/380 fluorescent ratio of fura2-AM cytosolic probe)) from Ctrl and CM pretreated with 200 µM of puromycin for 30 min. (**B**) Boxplots represent mitochondrial calcium content assessed by 25 µM of FCCP (Carbonyl cyanide 4-(trifluoromethoxy)phenylhydrazone) stimulation (expressed by the delta max of the 340/380 fluorescent ratio of fura2-AM cytosolic probe) in Ctrl condition and after 30 min of 200 µM puromycin pretreatment. (**C**,**D**) Mitochondrial Ca^2+^ rises recorded in 4mtD3cpv-positive CM electrically stimulated successively at 0.5, 1, and 2 Hz. (**C**) Representative time traces of mitochondrial Ca^2+^ concentration in Ctrl and after 30 min of puromycin pretreatment, expressed as YFP/CFP fluorescent ratio with a time binning of 4 (every 2.12 sec). (**D**) Corresponding averaged mean increase of mitochondrial Ca^2+^ concentrations at the same range of stimulation frequencies. (**E**) Representative time traces of calcein fluorescence from Ctrl, and 1 µM cyclosporin A (CsA)-pretreated and puromycin-pretreated CM. (**F**) Boxplots representing the slope of the mitochondrial calcein fluorescence decay induced by ionomycin stimulation. Data are represented as medians (except in E, mean ± SEM) from at least three independent experiments. *n* = cell count. Statistics: ++ *p* < 0.01, +++ *p* < 0.001 vs. Ctrl.

Next, we analyzed whether puromycin treatment could modify the cytosolic Ca^2+^ concentration ([Ca^2+^]_cyto_) using fura-2 loaded cells. Because acute puromycin perfusion failed to trigger a significant increase in [Ca^2+^]_cyto_, we submitted CM to a 30 min puromycin pretreatment, combined or not with emetine (as a control in order to inhibit puromycin activity). Steady-state [Ca^2+^]_cyto_ was unchanged after puromycin-pretreatment (fluorescent ratio: 0.4583 vs. 0.4577 in control (Ctrl)), but was slightly decreased after a pretreatment with emetine prior to puromycin (fluorescent ratio: 0.4453; Figure 2A,B). No change in total cell Ca^2+^ content, quantified thanks to ionomycin treatment, was observed in CM pretreated with puromycin or concomitantly pretreated by puromycin and emetine (Figure 3A–C). To explain these results, one should note that the time-course of the puromycin response via TLC is slower than the caffeine time-course (Figure 1B). Moreover, one should be reminded that cytosolic Ca^2+^ elevations are counteracted by the combined action of Ca^2+^ reuptake by SERCA pumps, extrusion by the plasma membrane Ca^2+^ ATPase (PMCA) and the plasma membrane Na^+^/Ca^2+^ exchanger (NCX), and absorption by the mitochondria [48]. Thus, Ca^2+^ leakage triggered by puromycin treatment did not change the global [Ca^2+^]_cyto_ at rest, even if TLC participated in its regulation (as shown by its inhibition by emetine prior to puromycin application). 

To complete the study of the putative dichotomy between the TLC Ca^2+^ store (activated by puromycin) and the RyR-dependent Ca^2+^ store (sensitive to caffeine) and to evaluate their relative size, we first perfused caffeine to deplete the RyR-dependent stores, and consecutively we applied ionomycin to reveal the residual reticular Ca^2+^ pool (Figure 2D–F). The caffeine response was similar in each condition: control, puromycin, and puromycin + emetine (Figure 2D,E), corroborating our previous [Ca^2+^]_r_ results (Figure 1). The size of the remaining caffeine-insensitive stores was smaller in puromycin-treated cells (fluorescent ratio: 0.005) than in controls (fluorescent ratio: 0.021; Figure 2F), proof of a prior depletion of TLC-dependent store. Conversely, emetine pretreatment not only abolished the effect of puromycin, but also triggered a larger increase (fluorescent ratio: 0.056) in the remaining Ca^2+^ stock compared to control CM (Figure 2F). This suggests that the inhibition of the reticular Ca^2+^ leak via TLC enhanced the concentration of this Ca^2+^ pool. Interestingly, we have previously shown similar results using human cancerous prostatic cells where the chronic inhibition of TLC caused an increase in the reticular Ca^2+^ concentration [25]. In order to sustain our results, we alternatively used pactamycin, another pharmacological Ca^2+^ leak inducer via TLC, and showed comparable effects on SR Ca^2+^ content (Figure S3).

It is common knowledge that reticular Ca^2+^ content is crucial for CM contraction, and changes in internal reticular Ca^2+^ stock could impair the excitation–contraction coupling mechanism. In order to evaluate whether TLC modulation could affect this parameter, we measured cytoplasmic Ca^2+^ transient in paced control and puromycin-pretreated CM (Figure 2G). Neither the rising slope (0.0033 vs. 0.0032 ΔF/F0.ms^−1^; Figure 2H), nor the amplitude (0.2923 vs. 0.3221 ΔF/F0; Figure 2I), nor the rate of decay (0.0102 vs. 0.0105 ΔF/F0.ms^−1^; Figure 2J) of Ca^2+^ transients were modified by puromycin pretreatment compared to the control, which goes along the line of two independent Ca^2+^ stores. 

In summary, our data illustrated that the well-known caffeine store cohabits with the brand new puromycin-sensitive store in primary mouse CM, which is in agreement with the work of Sleiman and colleagues [49], showing a dichotomy between cardiac endoplasmic reticulum (where protein synthesis takes place) and sarcoplasmic reticulum. Indeed, it would then be possible to have a fine [Ca^2+^]_r_ modulation independently of RyR pathways and by extension without modifying the excitation–contraction coupling. In future studies, it would also be interesting to look closer at the perinuclear level, a particular region enriched in both IP_3_R and ribosomes, and discover how TLC activation modulates the nuclear Ca^2+^ concentration.

During ischemia, the decline of ATP synthesis impairs SERCA activity. The consequences are cytosolic Ca^2+^ oscillations due to transient release of Ca^2+^ from the reticulum. We thus investigated whether puromycin preconditioning could attenuate cytosolic Ca^2+^ overload occurring during hypoxia in non-paced cells (Figure 2K). The average increase in the cytosolic Ca^2+^ concentration over hypoxia was figured out as the integrate of erratic Ca^2+^ transients above the basal cytosolic Ca^2+^ concentration. First, we visualized autonomous but erratic Ca^2+^ rises in cytosol of CM during hypoxia. Second, as reported in Figure 2L, the average increase in the cytosolic Ca^2+^ concentration due to hypoxia was found to be about two times higher than the one induced by caffeine (0.7783 s^−1^ and 0.4199 s^−1^, respectively). Third, the increase in the cytosolic Ca^2+^ concentration over a 30 min hypoxia was lowered by ≈64% in CM pretreated with puromycin compared to control CM (0.2829 s^−1^ and 0.7783 s^−1^, respectively). Lastly, caffeine-dependent Ca^2+^ stores after the 30 min hypoxia were similar between control and puromycin-preconditioned CM (Figure 2K,L). In addition, steady-state caffeine-dependent Ca^2+^ fluxes, estimated by the average increase in the cytosolic Ca^2+^ concentration after caffeine treatment, were significantly increased after 30 min of hypoxia (Figure S4). 

Taken together, our findings showed no effect of puromycin pretreatment on caffeine-dependent Ca^2+^ pool, nor, more importantly, on the excitation–contraction coupling. Furthermore, we demonstrated that the puromycin-mediated decrease in the Ca^2+^ stores was accompanied by a threefold lower increase in the cytosolic Ca^2+^ concentration during hypoxia. Finally, we found that the caffeine-sensitive Ca^2+^ store after hypoxia was similar in non-treated cells and in puromycin-treated CM, which substantiated our previous observations. 

### 3.2. Pharmacological Modulation of TLC with Puromycin Pretreatment Affected Mitochondrial Ca^2+^ Content in Beating (But Not in Resting) CM and Slowed down the mPTP Opening

As mitochondria buffer cytoplasmic Ca^2+^ increase and as mitochondrial Ca^2+^ uptake is faster than its release [50], there is probably a progressive enhancement of Ca^2+^ into the matrix during ischemia because the threshold of mPTP opening is reached, leading to apoptosis. We then looked at whether TLC activation could modify mitochondrial Ca^2+^ homeostasis. We thus wondered whether the puromycin pretreatment could shift the steady-state Ca^2+^ content in mitochondria of CM expressing the 4mtD3cpv FRET-based sensor (Figure S5A,B). No difference could be detected when comparing the control and puromycin-pretreated CM in the resting mitochondrial Ca^2+^ concentration ([Ca^2+^]_mito_; fluorescent ratio: 5.185 vs. 5.087, *p* = 0.1436; Figure S5B). However, the small decrease in the ratio of fluorescence after ionomycin treatment in the absence of external Ca^2+^ (Figure S5C) revealed that the steady-state Ca^2+^ concentration was at the edge of the dynamic range of 4mtD3cpv biosensor (K_d_ = 0.6 µM). This could have impaired the detection of significant differences in the mitochondrial Ca^2+^ content in control and puromycin-treated CM. We therefore performed an indirect measurement of the mitochondrial Ca^2+^ content. FCCP, an ionophore used to decouple ATP synthesis from the mitochondrial respiratory chain, induced the leak of mitochondrial Ca^2+^ content, which was measured by the cytosolic fura2-AM probe (K_d_ = 140nM). Puromycin pretreatment significantly decreased mitochondrial Ca^2+^ stores by ≈31% (fluorescent ratio: 0.0536 vs. 0.0781; Figure 3A,B), highlighting the role of TLC in the modulation of Ca^2+^ exchange between the reticulum and mitochondria.

We then measured mitochondrial Ca^2+^ fluxes in paced CM and assessed the effect of a puromycin pre-treatment (Figure 3C,D). Quantitatively, the increase of the frequency of stimulation was correlated to a steady-state mitochondrial Ca^2+^ elevation both in control and in puromycin-pretreated CM (two-way ANOVA, frequency stimulation affected the results with a *p*-value < 0.0001). Moreover, although [Ca^2+^]_mito_ rose with the pacing frequency in control CM, interestingly this rise was shifted to higher frequencies of pacing in CM treated with puromycin (two-way ANOVA, *p* = 0.0035; frequency–pretreatment interaction was F = 4.9, *p* = 0.0121). Because no difference could be detected in cytosol (Figure 2G–J), the apparent decrease in [Ca^2+^]_mito_ in beating cells could be explained by a direct effect of puromycin decreasing Ca^2+^ influx from reticulum to mitochondria. To test this hypothesis, we performed a Ca^2+^ retention capacity assay (CRC is the amount of Ca^2+^ that can be sequestered by mitochondria until the permeability transition occurs) in isolated mitochondria of CM and did not find any effect of puromycin pretreatment on either the Ca^2+^ intake or mPTP opening (Figure S6). These results suggest that puromycin neither directly challenged Ca^2+^ intake by MCU nor directly opened mPTP.

We next questioned if the puromycin-mediated decrease in mitochondrial Ca^2+^ content could retard mPTP opening in living CM. We valued mPTP opening by the “calcein/cobalt” method (Figure 3E,F). Cyclosporin A (CsA), known for slowing down mPTP opening, was used as a positive control [51]. We found that mPTP opening was significantly delayed in puromycin-pretreated (0.6665 compared to control 0.8559), whereas we only observed a tendency in CsA-pretreated CM (0.7287; *p* = 0.08). 

Jointly, our results point out that pharmaceutical activation of TLC enhances mitochondrial tolerance to handle Ca^2+^ by decreasing both steady-state mitochondrial Ca^2+^ content and the average Ca^2+^ level in mitochondria of beating CM. This may explain how puromycin pretreatment slowed down mPTP opening by decreasing reticular Ca^2+^ leak during ischemia and augmenting the tolerance to Ca^2+^ of mitochondria by reducing the average mitochondrial Ca^2+^ concentration. An enhanced tolerance of mitochondria to Ca^2+^ load has been revealed to be crucial to evade mPTP opening and in triggering cell death [51]. A similar proof-of-concept has been previously reported by Petrovski et al. [52], as they described the beneficial effects of thapsigargin (a SERCA pump inhibitor that induces reticular Ca^2+^ leak at low doses) in a preconditioning protocol in rats by decreasing infarct size and improving left ventricular function. However, it is obvious that SERCA inhibition will also lead to inhibition of CICR and thus prevent CM contraction [19].

### 3.3. Pharmacological Modulation of TLC with Puromycin Pretreatment Protected CM after In Vitro H/R, and Reduced Infarct Size in Mice Submitted to In Vivo I/R

To our knowledge, nothing is known about modulation of TLC activity during hypoxia or ischemia. First, one can assume that translation is reduced during hypoxia and it should be the same for the associated Ca^2+^ leak via TLC. Second, hypoxia triggers an increase in cytosolic Ca^2+^ concentration, partly due to a decline of ATP synthesis necessary for SERCA pumps to reuptake Ca^2+^ into the lumen. An interesting study characterized a calmoduline (CaM)-binding motif in Sec61α [36]. Using microsomes, the authors showed that CaM closes the channel in a Ca^2+^-dependent manner. Then, in the context of hypoxia, we may hypothesize that Ca^2+^ permeability of TLC is reduced.

**Figure 4:**
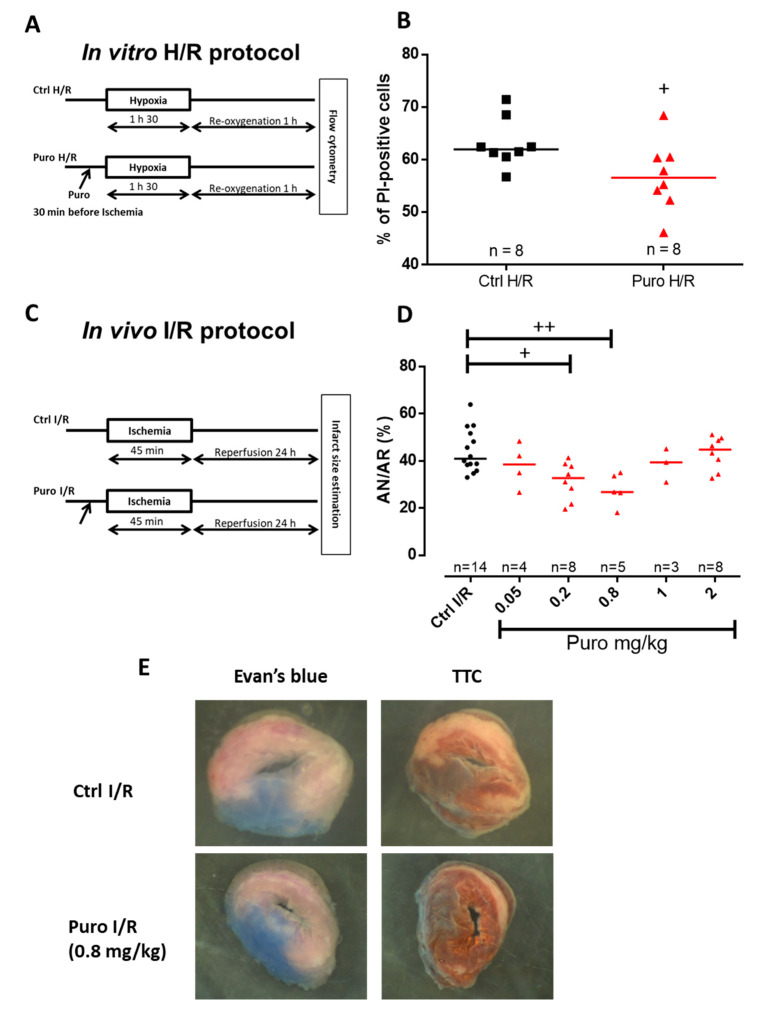
In vitro and in vivo CM protection by puromycin pretreatment after ischemia/reperfusion. (**A**) Experimental design representing ischemia-like hypoxia/reoxygenation (H/R) protocols achieved in isolated adult mouse CM. (**B**) Scatter plots showing mortality of CM subjected to H/R (Ctrl H/R) or CM concomitantly subjected to H/R and a 200 µM puromycin pretreatment (puro H/R). Evaluation of CM mortality was assessed via propidium iodide (PI) staining. (**C**) Experimental design showing the I/R protocols performed in mice by a blind test comparing different concentration of puromycin. (**D**) Scatter plots representing individual I/R mouse by the percentage of necrosis area (AN) over area-at-risk (AR). *n* = number of animals. Statistics: + *p* < 0.05, ++ *p* < 0.01 vs. Ctrl H/R. (**E**) Representative images of Evan’s blue- and triphenyltetrazolium chloride (TTC)-stained hearts from Ctrl I/R and Puro I/R mice.

In the following, we examined whether puromycin pretreatment (i.e., prior to hypoxia or ischemia) might be an effective cardioprotective strategy. CM death after hypoxia/reoxygenation (H/R) was appraised by a multilabeling flow cytometry analysis. Propidium iodide quantification was performed after 1 h 30 min hypoxia and 1 h reoxygenation with or without puromycin 200 µM pretreatment. Results highlighted a protective effect of puromycin by reducing cell mortality by ≈6% compared to control H/R (56% vs. 62%; Figure 4A,B). Concomitantly, CM were loaded with DilC1(5), MitoSOX Red, and MitoTracker Deep Red in order to respectively check the mitochondrial potential membrane, the production of ROS (reactive oxygen species), and the mitochondrial mass. No significant difference was observed between Ctrl and puromycin-treated cardiomyocytes submitted to H/R (Figure S7). This absence of effect on mitochondrial function meant that TLC activation did not worsen mitochondrial damage due to H/R but also did not improve it either, reinforcing our conclusions that TLC-conferred protection does not act directly on mitochondria but rather it is due to the modification of the reticular Ca^2+^ homeostasis.

Because TLC stimulation by puromycin enhanced cell survival when submitted to H/R, we assessed its putative protective action in an in vivo mouse model of myocardial I/R preconditioned or not with puromycin. In order to optimize the treatment efficiency, we performed dose–effect experiments. Infarct size estimation was figured out after 45 min ischemia and 24 h reperfusion (Figure 4C). Optimal preconditioning was obtained with a 0.8 mg/kg puromycin treatment and showed a reduction of ≈34.5% in infarct size compared to control mice (Figure 4D,E). Higher puromycin concentrations failed to protect mouse heart. It is important to notice that neither blood pressure nor heart frequency were modified at the optimal dose of 0.8 mg/kg puromycin (Figure S8).

In brief, these last results underlined the fact that CM death can be partly prevented during ischemia by a moderate reticular Ca^2+^ depletion via TLC activation, which in turn might decrease Ca^2+^ toxicity in both cytosol and mitochondria. One can easily imagine that TLC activation, prior to ischemia, increases the reticular Ca^2+^ leak. In turn, it might cause a mild ER stress, from which there is growing evidence that it contributes to protect CM from cell death [53].

## 4. Conclusions

Our results showed for the first time that the modulation of TLC-dependent reticular Ca^2+^ store is correlated with the modulation of several processes involved in cell fate—mitochondrial tolerance for cytoplasmic Ca^2+^ increase, mPTP opening, and tilting the cell balance on the side of cell survival. In fact, most studies on CM protection during ischemia/reperfusion relied on avoidance of mitochondria death and emphasized the concept of direct inhibition of mPTP. We believe that the multiple consequences of the decrease in reticular Ca^2+^ content could induce a robust cardioprotective effect, less prone to individual fluctuations. Even though our results rely on pre-activation of TLC, that is, I/R preconditioning, and cannot be used during myocardial infarction, this study opens new perspectives on Ca^2+^ modulation that could be useful for further investigations on postconditioning, thus broadening future therapeutic possibilities.

## Supplementary Materials

The following are available online at https://www.mdpi.com/2073-4409/9/5/1319/s1, Figure S1: TLC expression in CM. Figure S2: TLC activation by puromycin did not modify the IP3R-dependant reticular Ca^2+^ stock. Figure S3: Similar effect for puromycin and pactamycin of cellular Ca^2+^ concentration. Figure S4: Cytosolic Ca^2+^ caffeine-induced release in control condition and after hypoxia. Figure S5: Mitochondrial Ca^2+^ concentration measured with 4mtD3cpv. Figure S6: CRC measurement. Figure S7: Puromycin pretreatment did not modify the mitochondrial function in CM after in vitro hypoxia/reoxygenation (H/R) protocol. Figure S8: Puromycin treatment at 0.8mg/kg had no effect on blood pressure and heart frequency.
