# Supplementary material for: Acute Induction of Translocon-Mediated Ca2+ Leak Protects Cardiomyocytes Against Ischemia/Reperfusion Injury
Source: Cells. 2020 May 25;9(5):1319. doi: 10.3390/cells9051319 (PMC7290748; doi:10.3390/cells9051319)
Supplement: Supplementary file 1 [file cells-09-01319-s001.pdf]

Supplementary materials

1. SUPPLEMENTARY METHODS

1.1 Immunoblot and immunostaining

Samples were lysed in RIPA lysis buffer (1 %Triton X100, 0.5 % Sodium deoxycholat and in mM: 150 NaCl, 50 Tris, supplemented with 5 EDTA, 1 Na3VO4, 20 NaF, 1 DTT, 1 anti-protease cocktail (Sigma) and 1 phosphatases inhibitors (Sigma)). A precellys homogenizer at 4 °C (Precellys 24, Bertin technologies) was used for isolated CM. Supernatant were then centrifuged for 15 min 3000 G. The supernatant protein concentration was measured using BCA kit (Interchim) and 40 µg of protein were denatured then deposed in 4-20 % acrylamide gel for migration and blotted on nitrocellulose membrane by electro transfer (Trans-Blot Turbo Transfer, Biorad). The membrane is then saturated with 5 % bovine serum albumin in 0.1 % TBS tween mixture. Proteins were detected after labelling by specific primary antibodies. Primary antibodies were used at a dilution of 1/1000 in the above mixture with overnight incubation at 4 °C, while secondary antibodies were diluted at 1/10000 in the same mixture and incubated for 1 h at RT.

Revelation was obtained by addition of the reaction substrate ECL prime reagent (GE healthcare). Acquisition was performed with Bio-Rad Molecular Imager Gel Doc XR+ (Bio-Rad). ImageLab software was used for quantification (Bio-Rad). Primary antibody: Sec61-α (ab15575) was purchased from Abcam, α-Tubulin (sc-5286) was purchased from Santa Cruz. HRP conjugated secondary antibodies were purchased from GE Healthcare and Alexa Fluor conjugated secondary fluorescent antibodies from Life technologies.

1.2 Mag-fura2-AM experiments

Cellular permeabilization was required in order to remove the cytoplasmic probe: after loading, CM were washed with CCB, for 5 min. Following signal stabilization, 10 µM of digitonin (Sigma) was

added. As soon as the signal changed, extracellular medium was replaced by an intracellular medium (in mM: 25 NaCl, 125 KCl, 10 HEPES, 0.1 MgCl2, 0.5 CaCl2, 1 EGTA, 10 glucose, adjusted to pH7.4) supplemented by 200 mM ATP (Sigma). Drugs were added after signal stabilization.

1.3 Mitochondrial Isolation

Mice were euthanized by cervical dislocation, and the hearts were very quickly removed. Ventricles' tissues were minced on ice in isolation buffer A (in mM: 50 Tris, 1 EDTA, 70 saccharose, 210 mannitol, pH 7.4). They were cut roughly and homogenized in a glass-glass potter on ice. The ground material was then subjected to differential centrifugations at 4 °C. The first centrifugation was carried out at 1300 g for 3 min, allowing the cellular debris to be pushed. The supernatant was then recovered and centrifuged at 10000 g for 10 min, causing the sedimentation of the mitochondria. The pellet obtained was then resuspended in buffer B (same composition as buffer A without EDTA), and a final centrifugation was carried out at 10000 g for 10 min. The final pellet was then re-suspended in 100 µl of buffer B, and the mitochondrial proteins were finally quantified by Bradford assay.

1.4 CRC Measurement

Ca^{2+} retention capacities of 250 µg mitochondrial proteins, suspended in 2 ml incubation buffer (in mM: 50 KCl, 2 KH2PO4, 20 Tris, 150 sucrose, 5 succinate, pH 7.4), were measured at 25 °C using spectrofluorophotometers F-2500 and F-7000 digi lab Hitachi® equipped with magnetic stirring and thermostatic control. Extra-mitochondrial free Ca^{2+} was detected using 0,4 µM of Ca^{2+}-green-5N fluorescent probe, with excitation and emission wavelengths set at 500 and 530 nm respectively. Following a 2 min pre-incubation period, 20 nM of CaCl2 were added every 2 min until an increase in Ca^{2+}-green fluorescence was detected, indicating mPTP opening. Experiments were performed in the

presence and absence of puromycin (200 μM). The effect of the acute and the 30 min pre-treatment with puromycin were studied.

1.5 Probe multilabeling

Cells were loaded at 37°C 10 min before the end of the reperfusion phase, with DilC1(5), MitoSOX Red or MitoTracker Deep Red as previously described [46].

1.6 Arterial pressure and heart rate monitoring

Heart rate was measured using three 30G electrodes disposed subcutaneously at the anterior and posterior limbs of the animal. Data were retrieved via the iox2 software (Emka®) in a continuous mode. Puromycin pretreated mice were injected IV under anesthesia with the 0.8 mg/kg dose while control mice were injected with the same volume of saline solution 0.9 %.

Blood pressure was determined non-invasively at the animal's tail (Coda, KentScientific®). After IV injection of the 0.8 mg/kg of puromycin, mice were kept in contention in a cylinder provided for this purpose and positioned on a heating pad at 37 °C, while the cuff and the sensor are positioned at the base of the tail of the animal. The measurements are made every 5 min and the data are recorded directly by computer.

Habituation sessions were performed once or twice a day for several days upstream measurement day to reduce animal stress due to the restraint.

2. SUPPLEMENTARY FIGURES

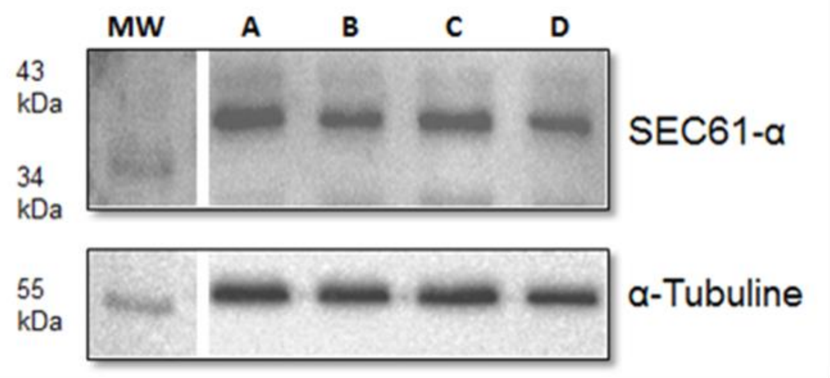

Figure S1: TLC expression in CM.
Sec61, the major component of TLC was detected by the immunoblotting of CM protein extracts from 4 mouse hearts by SDS-PAGE. α-tubulin reported the protein loading in each lane. 40 μg of proteins were loaded.

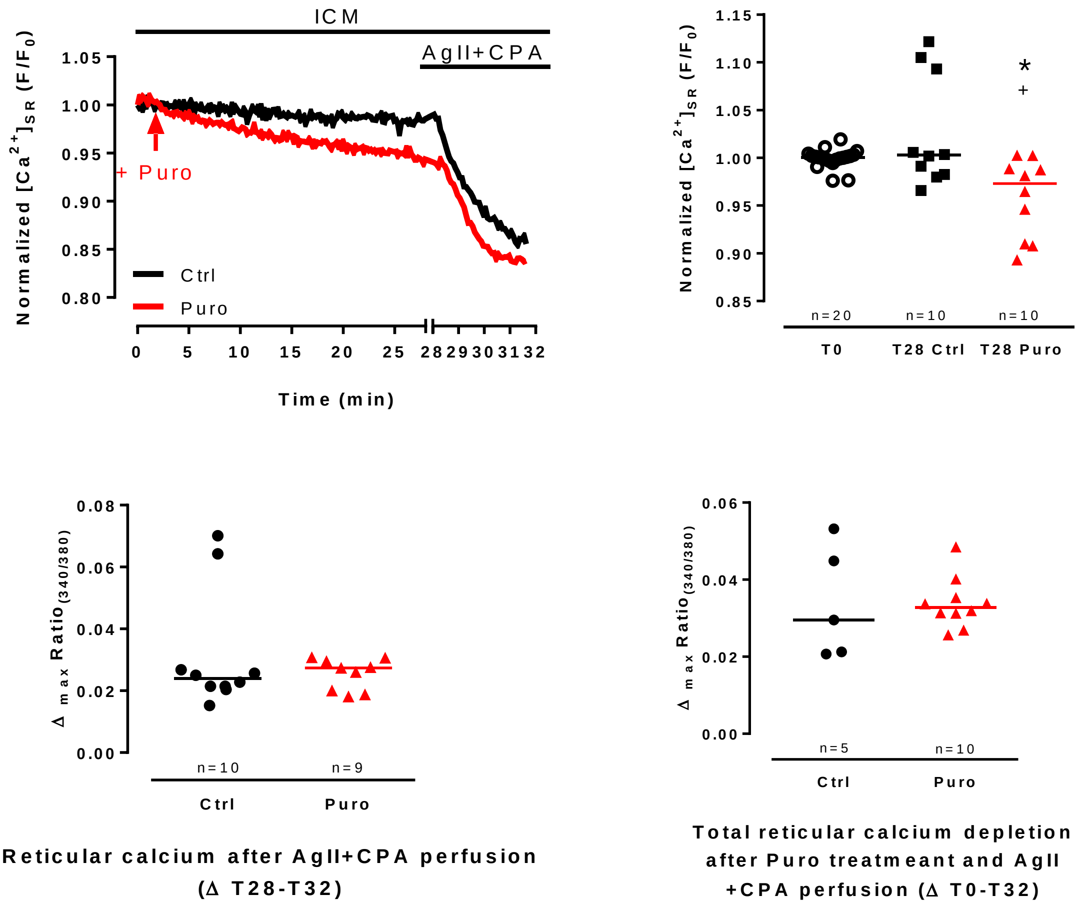

Figure S2: TLC activation by puromycin did not modify the IP3R-dependant reticular Ca^{2+} stock. (A) Graphical representation of the mag-fura2-AM fluorescence ratio evolution with time in control condition and in response to 25 min of 200 µmol/L puromycin treatment, both stimulated with 5 µM Angiotensin II (AgII)+15 µM CPA (inhibitor of SERCA pumps); Representative curve of reticular fluorescence evolution in Ctrl condition and under puromycin treatment. (B) Scatter plots of reticular Ca^{2+} decrease at the end of 25 min perfusion with or without puromycin compared to initial fluorescence. The mean of the Ctrl was used as reference (100 %). (C) The reticular Ca^{2+} stock after puromycin or Ctrl treatment estimated by AgII+CPA stimulation, calculated as difference between fluorescence level at stimulation time and final fluorescence. (D) The total Ca^{2+} decrease, calculated as difference between initial fluorescence and final fluorescence, under Ctrl or puromycin treatment followed by AgII+CPA stimulation. n=cell count. Statistics: + $p<0.05$, *vs.* T=0, * $p<0.05$ *vs.* T=28 Ctrl.

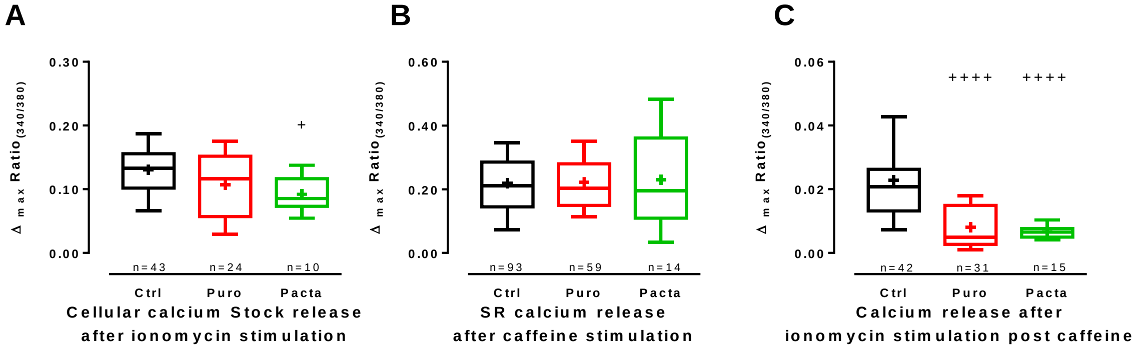

Figure S3: Similar effect for puromycin and pactamycin of cellular Ca^{2+} concentration

Ca^{2+} content was figured out by the maximum amplitude of fura2-AM fluorescence ratio (Δmax Ratio(340/380)) after the addition of different stimulations in comparison between Control, and pretreated CM with 200 µM puromycin or 1 µM pactamycin (pacta). (A) Boxplots representing the total cell Ca^{2+} content assessed by 5 µM of ionomycin stimulation. (B) RyR2-dependent Ca^{2+} stores assessed by 10 mM of caffeine stimulation and (C) remaining cell Ca^{2+} content after caffeine stimulation assessed by ionomycin stimulation. n=cell count. Statistics: ++++ $p<0.0001$, + $p<0.05$ vs. Ctrl, *** $p<0.001$ vs. Puro.

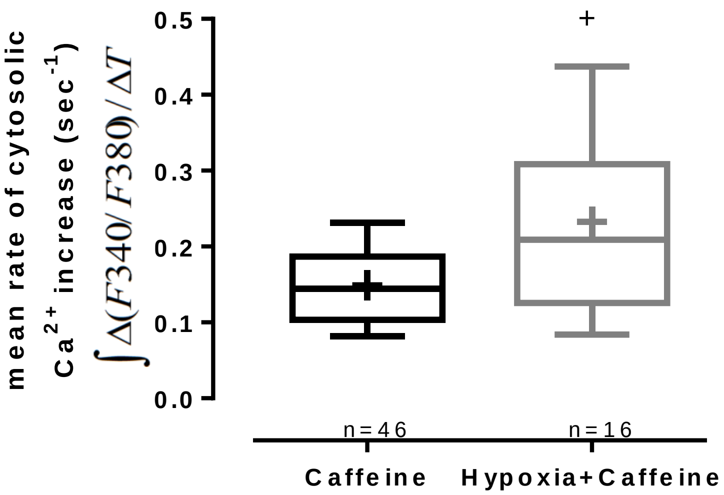

Figure S4: Cytosolic Ca^{2+} caffeine-induced release in control condition and after hypoxia. Boxplots showing average increase in the cytosolic Ca^{2} concentration ([Ca^{2+}]cyto) was after 30 min of ischemia-like hypoxia, as measured by the masses of fura2-AM fluorescence over time in CM stimulated by 10 mM of caffeine in Ctrl condition and after 30 min of ischemia-like hypoxia. n=cell count. Statistics: + $p<0.05$ unpaired t-test.

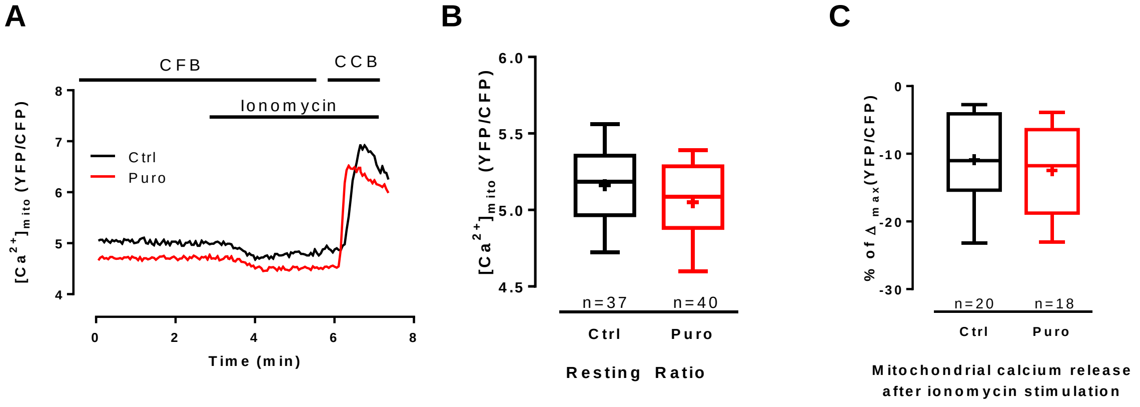

Figure S5: Mitochondrial Ca^{2+} **concentration measured with 4mtD3cpv.**

(A) Representative time traces of mitochondrial Ca^{2+} concentration (expressed as YFP/CFP fluorescent ratio) from Ctrl and CM pretreated with 200 μM of puromycin for 30 min. (B) Boxplots representing resting Ca^{2+} ration in mitochondria from Ctrl and puromycin pretreated cells. (C) Boxplots representing mitochondrial Ca^{2+}content after 5 μM of ionomycin stimulation in both Ctrl and puromycin pretreatment conditions.

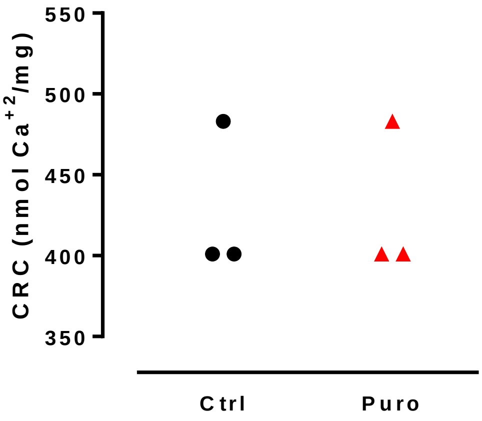

Figure S6: CRC measurement.

Scatter plots showing mean rates of Ca^{2+} concentration at which opening of the mPTP occurred. Concentration was calculated in the presence of succinate as a substrate for complex II. Isolated mitochondria from adult mice heart pretreatment with 200 µM puromycin has no significant effect on their Ca^{2+} retention capacity (CRC). Experiments were repeated 3 times.

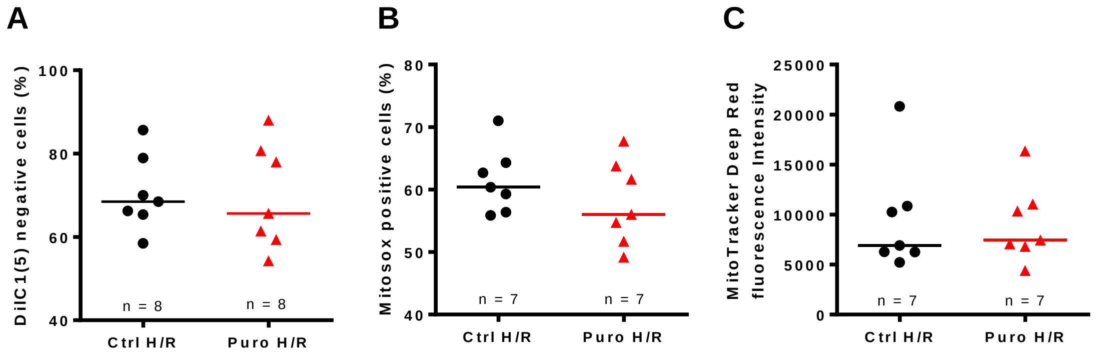

Figure S7: Puromycin pretreatment did not modify the mitochondrial function in CM after *in vitro* hypoxia/reoxygenation (H/R) protocol.

Scatter plots representing: (A) mitochondrial membrane potential (DilC1(5)), (B) reactive oxygen species production (MitoSOX Red), and (C) mitochondrial mass (MitoTracker Deep Red). Wilcoxon matched-pairs signed rank tests were performed.

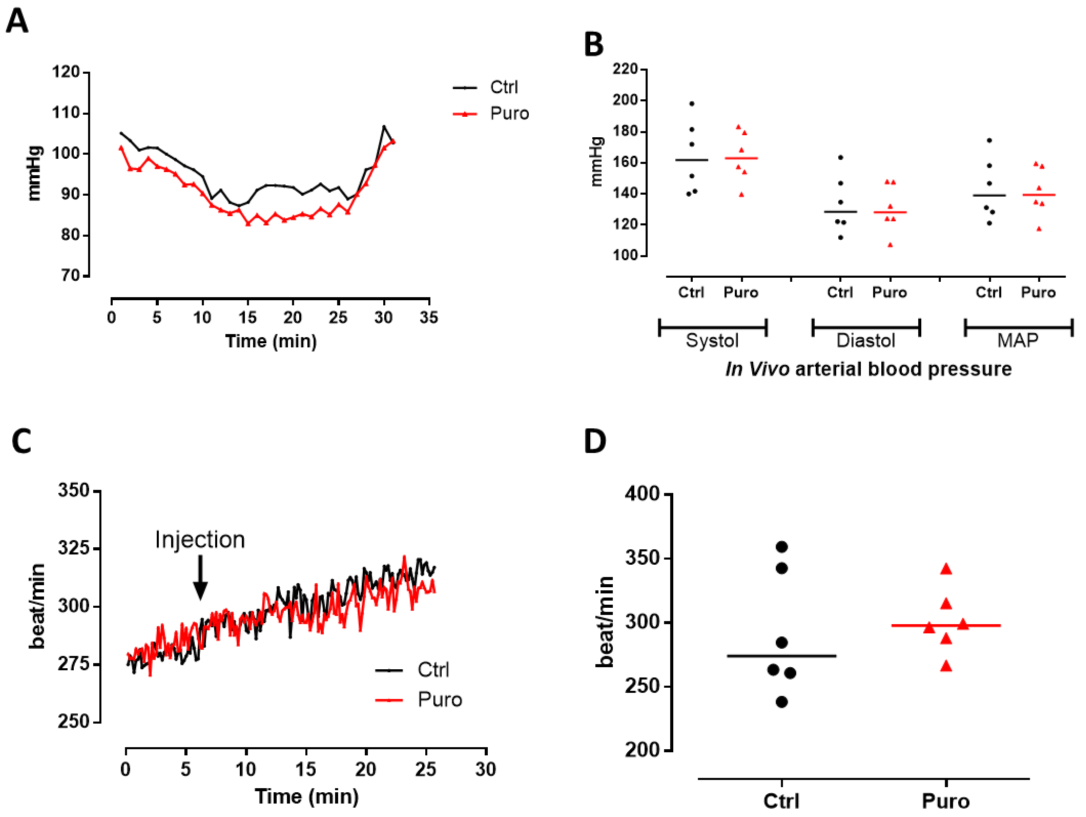

Figure S8: Puromycin treatment at 0.8mg/kg had no effect on blood pressure and heart frequency. (A) Time traces of the mean arterial pressure (MAP) calculated by the formula: (MAP = 1/3 (Systolic blood pressure – Diastolic Blood Pressure) + Diastolic Blood Pressure) during 30 min after 0.8 mg/Kg pyromycin IP injection compared to Ctrl. (B) Scatter plots representing difference in blood systolic, diastolic and MAP between Ctrl and 0.8 mg/kg puromycin pretreated mice. (C) Time traces of Ctrl mice heart compared to 0.8 mg/kg puromycin pretreated mice during 30 min (D) Scatter plots of the different measured heart rate mean of Ctrl mice in comparison to 0.8 mg/kg puromycin pretreated mice. n=6 mice in each group.
